# Multidrug resistant commensal Escherichia coli in animals and its impact for public health

**DOI:** 10.3389/fmicb.2013.00258

**Published:** 2013-09-03

**Authors:** Ama Szmolka, Béla Nagy

**Affiliations:** Institute for Veterinary Medical Research, Centre for Agricultural Research, Hungarian Academy of SciencesBudapest, Hungary

**Keywords:** commensal, *E. coli*, antimicrobial resistance, resistance genes, resistance mechanisms

## Abstract

After the era of plentiful antibiotics we are alarmed by the increasing number of antibiotic resistant strains. The genetic flexibility and adaptability of *Escherichia coli* to constantly changing environments allows to acquire a great number of antimicrobial resistance mechanisms. Commensal strains of *E. coli* as versatile residents of the lower intestine are also repeatedly challenged by antimicrobial pressures during the lifetime of their host. As a consequence, commensal strains acquire the respective resistance genes, and/or develop resistant mutants in order to survive and maintain microbial homeostasis in the lower intestinal tract. Thus, commensal *E. coli *strains are regarded as indicators of antimicrobial load on their hosts. This chapter provides a short historic background of the appearance and presumed origin and transfer of antimicrobial resistance genes in commensal intestinal *E. coli* of animals with comparative information on their pathogenic counterparts. The dynamics, development, and ways of evolution of resistance in the *E. coli* populations differ according to hosts, resistance mechanisms, and antimicrobial classes used. The most frequent tools of *E. coli* against a variety of antimicrobials are the efflux pumps and mobile resistance mechanisms carried by plasmids and/or other transferable elements. The emergence of hybrid plasmids (both resistance and virulence) among *E. coli* is of further concern. Co-existence and co-transfer of these “bad genes” in this huge and most versatile *in vivo* compartment may represent an increased public health risk in the future. Significance of multidrug resistant (MDR) commensal *E. coli* seem to be highest in the food animal industry, acting as reservoir for intra- and interspecific exchange and a source for spread of MDR determinants through contaminated food to humans. Thus, public health potential of MDR commensal *E. coli* of food animals can be a concern and needs monitoring and more molecular analysis in the future.

## INTRODUCTION

### THE ANIMAL AND HUMAN FACE OF ANTIMICROBIAL USE: OVERLAPS AND DIFFERENCES

Over the past half century, the use of antimicrobials to treat infections in human and animals has generated an enormous antimicrobial pressure not only on targeted pathogens but also on commensal bacteria. As a response to therapeutic antimicrobial pressure, the intestinal flora may undergo dramatic changes, including reductions in the orders of *Bifidobacteriales, Clostridiales, Campylobacterales*, but an exponential and sudden increase of *Enterobacteriales* (*Escherichia*) and *Lactobacillales* (*Enterococcus*) as described very recently in case of streptomycin and/or tetracycline therapy of laying hens (Videnska et al., 2013). The accumulating effect of traditional antimicrobials was completed by the continuous discovery and introduction of new therapeutical drugs, which drives bacteria to be trained to constant changes by selecting appropriate antimicrobial resistance pheno- and genotypes. Once armed with the required set of antimicrobial resistance genes, bacterial strains may have the advantage to survive and spread both in animal and human populations, since with few exceptions the same antimicrobial classes are used to treat infections in animals and humans (Guardabassi and Courvalin, 2006).

Although antimicrobial classes are common in veterinary and human medicine, their importance may vary according to the species and application. Majority of the antibacterial compounds are generally used to treat a wide range of animals and infections, but there are drugs with applications restricted to certain groups of species (e.g., difloxacin to avian infections).

On the other hand, some antimicrobial classes such as cephalosporins (first to fourth generation) are represented by a large number of compounds for treating serious infections in humans, while only few of them has veterinary application (World Organisation for Animal Health [OIE], 2007; World Health Organization [WHO], 2007). In addition, based on their importance in medication, availability of alternatives, selection of cross-resistance, and frequency of use, antimicrobials are ranked into critically-, or highly important drugs. Obviously, these categories do not necessarily overlap.

There are further remarkable differences in the use of antibacterial agents in humans and animals, especially in food animals. In humans drugs are generally administered directly to sick individuals, in contrast to food animals, where usually (groups of animals) are affected at the same time. Moreover, in animal husbandry drugs are used not only for therapeutic purposes, but also for prophylaxis and – earlier – as growth promoters, in subtherapeutical concentrations. The latter group was represented among others by members from glycolipids (bambermycin), glycopeptides (avoparcin), polypeptides (bacitracin), quinoxalines (olaquindox), and tetracyclines. Although the use of growth promoters has been banned in the European Union from 2006, some of them are still used in other regions (penicillins, tetracyclines – USA; chloramphenicol, fosfomycin – Asia; Guardabassi and Courvalin, 2006).

Beside the large overlaps in the use of antimicrobials in animals and humans, the above listed differences may result increasing incidence and changing patterns of antimicrobial resistance not only among pathogenic *Escherichia coli* from animals and humans, but even among their commensal counterparts derived from a human and from a wide range of animal species.

For this review commensal *E. coli* are defined as bacteria isolated from healthy animals without known virulence (toxic, adhesive, invasive) attributes playing a role in a specific disease caused by *E. coli*. The dynamics, development, and ways of evolution of resistance traits in *E. coli* populations may differ according to the hosts, and antimicrobials used (Szmolka et al., 2012). This review intends to discuss the current knowledge on antimicrobial resistance in commensal strains of *E. coli*, with special regard to those from food animals.

However, the known complexity, together with the changing mechanisms of resistance in *E. coli*, and the reduced information regarding possible virulence and fitness genes of commensal strains, do not allow a strict separation of this group from the clinical/pathogenic *E. coli*. Therefore we will focus mainly to the “innate” and acquired resistance mechanisms frequently characterizing *E. coli* strains from healthy animals with considerations of their multidrug resistance (MDR). Furthermore, special emphasis will be addressed to novel resistance mechanisms recently affecting also the commensal strains of *E. coli*, and to the combination and/or co-transfer of resistance and virulence traits, which raises particular concern in the therapy of infectious diseases.

## MULTIDRUG RESISTANCE IN COMMENSAL *E. coli*

Due to the introduction of antimicrobials as growth promoters and/or as therapeutic agents combating bacterial infections, targeted pathogenic *E. coli* strains and their commensal counterparts – habitating the intestine – are similarly exposed to the effect of various antimicrobial compounds, thereby being forced to develop different strategies to survive and grow in the newly established toxic environment. The most efficient and sophisticated defense mechanism is the acquisition of MDR, characterized by the complex interaction of different mechanisms (e.g., drug efflux, enzymatic inactivation, target protection) conferring simultaneous resistance to a wide range of older and/or new antimicrobial compounds or drug classes.

Recently, MDR became widely established especially in Gram-negative bacteria such as *E. coli*, being a “versatile” species encompassing different pathotypes, but also as a member of the normal intestinal flora. Therefore commensal *E. coli* may play a special role in the accumulation and interplay between resistance traits.

In contrast to pathogenic strains, which are in the focus of the therapy, commensal strains are generally marginalized in many respects, due to their reduced clinical significance. Tackled as potential reservoirs of resistance determinants, the prevalence of antimicrobial resistance in commensal *E. coli* from food animals is monitored regularly (European Food Safety Authority and European Centre for Disease Prevention and Control [EFSA and ECDC], 2010). However, their genetic attributes, such as the co-existence and spread of resistance genes, and their ability to colonize the human intestine are not adequately considered.

### COMPLEXITY AND TRANSFERABILITY OF ANTIMICROBIAL RESISTANCE MECHANISM

In the constantly changing battle against antimicrobials, pathogenic and commensal bacteria learned to develop or acquire appropriate weapons, and consequently MDR proved to be a perfect tool in this continuous fight for survival. Similar to other members of *Enterobacteriaceae*, *E. coli* can choose from several mechanisms to fend off the simultaneous effect of various antimicrobial agents.

Certain protein structures, which mediate the simultaneous efflux of a wide range of antimicrobials from the cells, or cause decreased membrane permeability are parts of ancient, mostly chromosomally encoded mechanisms causing MDR in different *E. coli* populations.

The co-existence of multiple individual resistance mechanisms in different combinations (e.g., efflux and ribosomal target protection mediating resistance to the same drug class) promotes the selection of MDR strains and confers elevated level of resistance at the same time. The majority of resistance genes encoding a wide variety of resistance mechanisms are carried by mobile genetic elements such as plasmids, transposons, integrons (Iyer et al., 2013) which favors the co-transfer of MDR phenotypes between commensals and pathogens, animals and humans.

### MULTIDRUG RESISTANCE IN ONE STEP: MAJOR “INNATE” EFFLUX SYSTEMS FOR THE ACTIVE REMOVAL OF ANTIMICROBIALS IN *E. coli*

The energy-dependent extrusion of antimicrobials is an ancient and widely used key mechanism thought to mediate “innate MDR” in Gram-negative bacteria. This type of MDR represents a major concern, because a single species of multidrug efflux pump can confer simultaneous resistance to a wide range of antimicrobials. Thus it is not surprising, that the structure, the mechanism as well as the expression regulation of different efflux systems are extensively studied recently in diverse bacteria of clinical and zoonotic potential, including *E. coli* (Li and Nikaido, 2009; Nikaido, 2009).

Among the large structural and functional diversity of the proton-dependent efflux machineries, – including members of the major facilitator superfamily (MFS), the small MDR (SMR) family, and the resistance-nodulation-division (RND) superfamily – the archetypal AcrAB–TolC transporter (RND superfamily) is of prime importance mediating MDR in *E. coli* (Li and Nikaido, 2009). This ability was clearly demonstrated by constructing mutants of a laboratory *E. coli* strain with deletions of individual pump genes or gene groups according to the pump family. In contrast to other pump families, deletions of *tolC* or *acrAB* encoding different transport proteins of the above three-component machinery resulted the greatest increase in susceptibility for a broad range of compounds tested, including diverse drug classes of clinical importance, such as: penicillins, phenicols, macrolides, quinolones, tetracyclines (Sulavik et al., 2001).

Although no similar studies have been performed to reveal the contribution of MDR efflux in non-pathogenic strains, we think that the above finding may reflect a similar scenario in commensal *E. coli* from the “pre-antibiotic era.” It seems that the commensal *E. coli* strains are not yet fully adapted to the high antimicrobial pressure of the recent years.

The wide substrate range represents only a partial advantage as the MDR efflux pumps generally confer low level (subclinical) resistance. However, bacteria seem to overcome this “limitation” by tricky combinations of defense systems. A good example to this comes from the outer membrane channel protein TolC of the AcrAB–TolC efflux apparatus, which can work together with transporters from other efflux pump families. The functional interplay of TolC with MdfA in *E. coli* as representant of the MFS or with ErmE from the SMR family does not only extend the substrate range, but also increase the transport efficiency, in the final removal of drugs from the periplasm into the surrounding medium (Lee et al., 2000). Moreover, efflux generally acts synergistically with other resistance mechanisms encoded either by the chromosome or being acquired via different transferable elements, to provide MDR of clinical relevance in both pathogenic and commensal strains of *E. coli*.

### RESISTANCE GENE ASSOCIATIONS AND CORRESPONDING MECHANISMS ACCOUNTING FOR MDR IN COMMENSAL *E. coli* FROM ANIMALS

The first important step in predicting the food safety, animal- and public health significance of antimicrobial resistance is to describe associations between different resistance features being distributed among commensal *E. coli* in food animal production. The complex nature of these relations comes from the enormous diversity of chromosomally encoded and/or acquired resistance mechanisms, which were further expanded in the recent years by a number of novel resistance mechanisms with special interest.

Our understanding on the zoonotic significance of MDR *E. coli* circulating in the food chain could be enhanced by comparative characterization of MDR commensal *E. coli* strains derived from food animals. However, the comprehensive overview of the most prevalent resistance traits in the light of their MDR nature, raises two main difficulties: (i) the resistance phenotypes are usually not confirmed by underlying genes/mechanisms, which prevent to characterize resistance in its whole complexity, (ii) in general, few data are provided on genetic associations accounting for MDR pheno-/genotypes.

**FIGURE 1:**
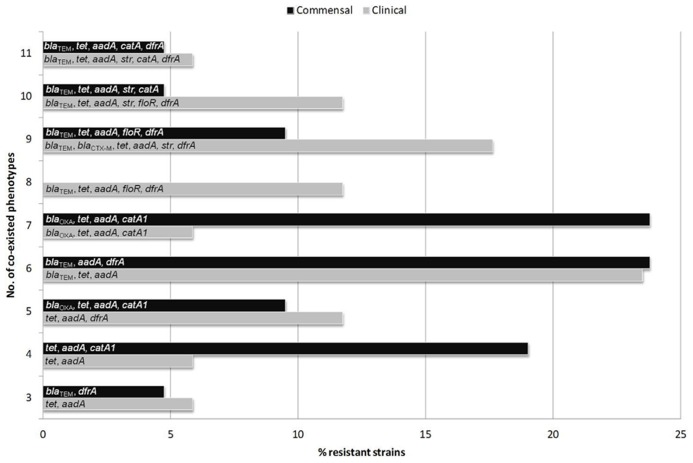
**Resistance gene associations identified in multidrug resistant commensal and clinical *E. coli* strains from food animals, grouped according to increasing numbers of co-existed resistance phenotypes.** Class 1 integrons (*intI1)* were detected in all groups of MDR commensal and clinical strains while class 2 integrons (*intI2)* were carried in groups with 5 and 9 co-resistant phenotypes of clinical, and in the group with 6 co-resistant phenotypes of commensal *E. coli* strains.

To overcome the above deficiencies we will focus on reviewing the common resistance traits of commensal *E. coli* from food animals as mirrored by recent findings relevant to the above considerations. With this approach in mind, a comprehensive pheno- and genotyping of resistance and virulence features was performed recently on a selected *E. coli* strain collection of poultry, pig, and bovine origin, representing diverse sample sources from healthy and sick animals (Szmolka et al., 2012). It was found, that regardless of the host source, resistance genes were abundantly present to confer simultaneous resistance to three or more antimicrobial compounds/classes (**Figure 1**). Results indicated the persistence of a common “multiresistance pattern,” represented by associations between several important antimicrobial classes (and corresponding genes) including aminoglycosides (*aadA1*-like and *strA/B*), β-lactams (*bla*_TEM_), sulfonamides (linked mainly to class 1 integrons – *intI1*), and tetracyclines [*tet*(A) and *tet*(B)] is especially distributed and maintained in animal husbandry (**Figure 1**). Such resistance patterns could be tackled as characterizing *E. coli* strains from clinical settings as well (Szmolka et al., 2012).

The widespread dissemination of the same multiresistance phenotypes mediating resistance to “older” antimicrobials was also reported by consecutive European surveys, conducted for monitoring of antimicrobial susceptibility in *E. coli* isolates from healthy food animals (Bywater et al., 2004; European Food Safety Authority and European Centre for Disease Prevention and Control [EFSA and ECDC], 2010; de Jong et al., 2012). Similar to this, the association between ampicillin–doxycicline–tetracycline–sulfamethoxazole/trimethoprim resistance phenotypes was reported to be predominant in poultry and swine production in China (Jiang et al., 2011).

Quantitative differences can be detected according to the host species and geographical regions as follows: the prevalence of resistant *E. coli* strains in cattle was lower than in pig and poultry (Guerra et al., 2003a; Kojima et al., 2009), and the lowest level of resistance was registered in Northern European countries (European Food Safety Authority and European Centre for Disease Prevention and Control [EFSA and ECDC], 2010). This may be a consequence of diverse farming conditions (pig and poultry are much more intensively housed and treated against infections) and country-specific practices and regulations for the use of antimicrobials. In addition to this, the influence of animal age and the purpose of production (e.g., dairy or beef) type may also be also considered (Berge et al., 2010).

The molecular characterization of underlying resistance determinants reveal that common co-resistant phenotypes of animal *E. coli* are based on certain genes: ampicillin (*bla*_TEM-1_-like), streptomycin/spectinomycin (*aadA1*-like and *strA/B*), tetracycline [*tet*(A) and *tet*(B)], sulfamethoxazole (*sul1*), and trimethoprim (*dfrA1*-like). Beside these major genes listed, several additional genes encoding the same resistance phenotype were identified: *bla*_OXA-1_-like (ampicillin), *sul2* (sulfamethoxazole), *dfrA14* and *dfrA17* (trimethoprim; Guerra et al., 2003a; Bonnet et al., 2009; Szmolka et al., 2012). Of known, these genes are often related to mobile genetic elements (discussed below), which imply the circulation of certain conserved genetic vectors in food animal *E. coli* populations.

In contrast to the above data on food animals, our knowledge about antimicrobial resistance of commensal *E. coli* in companion animals and non-captive wild animals is much more limited. Companion animals (especially dogs and cats) started receiving increased attention only during the last 10 years. Although most of these studies have been focusing mainly on Gram-positive microbes (Guardabassi et al., 2004), some recent data are primarily available on phenotypic resistance traits of commensal *E. coli* as well. An interesting case study on MDR commensal *E. coli* from cohabitant pets, humans, and their household with the same antimicrobial resistance patterns indicated direct contacts and cross contamination between all cohabitant species including humans (Martins et al., 2013).

Wildlife studies have also shown several examples of antimicrobial resistance of commensal *E. coli*. One of the first descriptions of such strains in wild mammals were given by Costa et al. (2006) reporting the presence of extended spectrum β-lactamases CTX-M, TEM, and SHV classes (Jacoby and Munoz-Price, 2005). Studies focusing on wild boars reported class 1 and class 2 integrons with their usual resistance gene cassettes in Poland (Mokracka et al., 2012), or an additional range of extended-spectrum β-lactamases (ESBL) and nalidixic acid and ciprofloxacin resistance determinants in Portugal (Poeta et al., 2009). Similar findings were reported on commensal *E. coli* from a wide range of wild mammals in Czech Republic/Slovakia (Literak et al., 2010). Similarly, an approximately 5% of different wild birds representing different regions proved to be carrying MDR *E. coli* in Germany (Guenther et al., 2010), with the most frequent phenotypic resistances being ampicillin, tetracycline as supported by minimal inhibitory concentration (MIC) values. A recent study focusing on Portuguese buzzards showed that >50% of commensal *E. coli* of these animals could be reservoirs of a wide range of antimicrobial resistance genes and of class 1 integrons. The tested *E. coli* isolates showed high levels of resistance to streptomycin and tetracycline, and the resistance genes detected were: *bla*_TEM_, *tet*(A) and/or *tet*(B), *aadA1*, *cmlA*, *aac(3)-II*. *sul1* and/or *sul2* and/or *sul3*. Besides, virulence-associated genes *papC* (P fimbriae) and *aer* (aerobactin) were present in >10% of these fecal isolates (Radhouani et al., 2012). A European study of Literak et al. (2012) on wintering omnivorous rooks commonly (37%) found fecal samples with ciprofloxacin resistant *Enterobacteriaceae* isolates carrying plasmid-mediated quinolone resistance (PMQR; overwhelmingly *qnrS1*) genes. Based on above observations, it seems that commensal *E. coli* of wildlife can be interpreted as indicators of contamination from the environment and represent less risk of exposure for human health.

In retrospect, the emergence and spread of the above common multiresistance pattern leads back into the 1970s (Tadesse et al., 2012), and demonstrates a perfect correlation with the approval and introduction of corresponding drugs in clinical use (Schwarz et al., 2006). As a consequence of the long term and intensive use of these broad spectrum antimicrobial agents alone or in combinations (e.g., sulfamethoxazole/trimethoprim) and of the introduction of newer compounds in human-, and veterinary medicine, the prevalence of *E. coli* strains with high number of clinically important co-resistant phenotypes in man and animals shows an increasing trend over time (Tadesse et al., 2012).

### ADDITONAL IMPORTANT RESISTANCE FEATURES OF ANIMAL *E. coli*

Beside the above described multiresistance pheno- and genotypes identified among commensal *E. coli* strains from healthy food animals, some additional phenotypes with MDR potential should also be considered. In comparison with the former group, these are overall less prevalent and in some extent show host-related distribution.

The extended use of (fluoro)quinolones to treat poultry infections lead to the increase of quinolone resistance among *E. coli* strains in poultry industry. Although quinolone resistance is in general not highly frequent in animal farming (Guerra et al., 2003a; Kojima et al., 2009), but high level of nalidixic acid resistance (67%) and moderate resistance (29%) to ciprofloxacin was reported in broiler chickens (Kmet and Kmetová, 2010). Significant differences in incidence of ciprofloxacin resistance was observed in turkeys (breeding or meat flocks; Gosling et al., 2012).

The sequence analysis of the chromosomal *gyrA* and *parC* mutations encoding DNA gyrase and topoisomerase IV identified the classical genotype of Ser83→Leu and/or Asp87→Asn (*gyrA*) and Ser80→Ile (*parC*) being the main mechanism responsible for quinolone resistance in commensal and clinical *E. coli*. The level of resistance show correlation with the number of mutations produced (Guerra et al., 2003a; Kmet and Kmetová, 2010).

Overall, low to moderate incidence of chloramphenicol resistance is registered in food animals of Europe (European Food Safety Authority [EFSA], 2009), despite the fact that this drug was not allowed for use in food animals after 1994. However, its derivative florfenicol is licensed for treatment of respiratory infections in cattle and pigs. The identification of resistance genes behind certain phenotypes reveal that in food animal *E. coli* strains resistance to chloramphenicol is mediated mostly by the genes *catA1, floR*, and *cmlA1* (Guerra et al., 2003a; Glenn et al., 2012; Szmolka et al., 2012) responsible for the enzymatic inactivation and the active extrusion of the drug, respectively. The *catA1* gene is often integrated within a resistance gene cluster, which ensure the persistence of corresponding resistance even in the lack of drug administration. This event may provide an explanation for the nascent resistance to chloramphenicol and will be discussed below.

Finally, the elevated incidence of gentamicin resistance in chicken and pig *E. coli* strains in eastern countries (Choi et al., 2011) may justify to be mentioned as potentially important resistance feature of *E. coli* from farm animals. Its significance is further increased by the consideration, that the presence of this phenotype was found to be associated with MDR in commensal and clinical and isolates of *E. coli* (Szmolka et al., 2012).

In addition to gene *aacC2* [aac(3)-II] which is though to be a marker gene coding for gentamicin resistance phenotype (Ho et al., 2010), several other types of aminoglycoside resistance genes, such as *aacC4* [*aac(3)-IV*], *aacC3* [*aac(3)-III*], and *aadB* [*ant(2”)-I*]) were infrequently identified in resistant strains (Guerra et al., 2003a; Choi et al., 2011). All of them mediate the enzymatic inactivation of the drug.

The high prevalence of certain co-resistance phenotypes is the best evidence to the co-selection and conservation of combined resistance mechanisms in commensal *E. coli*, reflecting the effect of irresponsible use of antimicrobials during the fight against pathogenic counterparts. The complex nature of these interactions is evident, since the interplay of different mechanisms, including the efflux-, enzymatic inactivation of the drug and the target protection are to be considered even in relation to individual phenotypes. Therefore, the molecular background of these resistances is not intended to be discussed here, and there are a number of comprehensive reviews to rely on (Sandvang and Aarestrup, 2000; O’Brien, 2002; Aarestrup, 2006; Schwarz et al., 2006).

Additionally, in the corresponding chapter special attention will be devoted to the newly discovered resistance mechanisms and gene assemblies, being developed as an adequate response to the introduction and extensive use of new drugs of critical importance especially in human medicine.

### ASSOCIATIONS BETWEEN ANTIMICROBIAL RESISTANCE AND VIRULENCE DETERMINANTS OF *E. coli* IN ANIMALS

Other important, but less well-studied associations are the ones of antimicrobial resistance and virulence in *E. coli*. This important question has been mostly studied on human pathogenic extraintestinal strains so far, and there are no direct evidences for co-transfer of resistance and virulence traits among commensal *E. coli* strains of humans. However, the co-selection of antimicrobial resistance and virulence genes is frequently observed among pathogenic *E. coli* strains, which can almost be regarded as self explanatory phenomena (Da Silva and Mendonça, 2012). A well-known example for this possible co-selection of CTX-M-15 resistance and virulence are provided by the ST131 clone of human ExPEC O25:H4-ST131 strains characterized mostly by higher virulence in murine models (Clermont et al., 2008).

For animal pathogenic *E. coli* there are also findings on such possible associations. A very recent example for this event, is provided by the fully sequenced large conjugative hybrid plasmid pTC of porcine enterotoxigenic *E. coli* (ETEC), possessing *sta* and *stb* genes for heat stable enterotoxins embedded in a toxin-specific locus (TSL), and a Tn*10* composite transposon carrying the tetracycline resistance gene *tet*(B) (Fekete et al., 2012). Interestingly this plasmid showed a high degree of similarity (98%) with the NR1 (DQ364638.1) and pC15-1a (AY4580161.1), the latter already mentioned above as a CTXM-15 carrying MDR plasmid associated with Canadian nosocomial extraintestinal *E. coli* infections (Boyd et al., 2004).

One example that we could come up at present for an animal commensal (intestinal) *E. coli* plasmid carrying both antimicrobial resistance and virulence determinants, has been described by Nógrády et al. (2006). This was a conjugative large (~120 kb) plasmid carrying a 1.6 kb class 1 integron (*dfrA1-aadA1*), and* tet*(A) resistance genes together with the gene *iss* (for increased serum survival). Other examples for concomitant occurrence of resistance and virulence genes has been described on avian ExPEC plasmids (pAPEC-O103-ColBM) carrying a MDR-encoding island and a ColV pathogenicity island (Johnson et al., 2010), and several other RepFIB/FIIA type hybrid plasmids showed the ability to acquire both virulence and MDR traits (Johnson and Nolan, 2009). Furthermore, Bonnet et al. (2009) reported that the MDR plasmid pAPEC-O2-R was characteristic to commensal *E. coli* strains from broilers raised on a diet supplemented with antimicrobials simulating farm conditions, and provided indirect evidence about simultaneous presence of resistance and virulence genes in these strains. A further finding on this line was provided by Szmolka et al. (2012) describing a strong correlation of gene *tet*(A) with the virulence genes *iroN* and *iss *in both commensal and extraintestinal avian *E. coli, *with indications that they might be similarly co-transferred on the same plasmids in both groups.

Regarding simultaneous occurrence and occasional co-transfer of resistance and virulence determinants among intestinal (commensal) and extraintestinal (pathogenic) isolates of human and avian *E. coli* strains, we should keep in mind that a clear distinction between extraintestinal pathogens and harmless commensals is not always easy (Tóth et al., 2012; Leimbach et al., 2013). Both groups are part of the normal intestinal flora and there is a broad spectrum of fitness and virulence between harmless commensal, and extraintestinal pathogenic strains of *E. coli* of man and of different avian species, which would probably be also true for some other food animals as well.

### MOBILE GENETIC ELEMENTS IN THE TRANSFER OF RESISTANCE IN COMMENSAL *E. coli*

The observation that a high number of same or closely related resistance genes are constantly circulating between bacteria of different species or even genera lead to the recognition, that the horizontal gene transfer represents the most effective tool in the acquisition and widespread dissemination of multiresistance pheno- and genotypes (Ochman et al., 2000). Clustering several resistance genes on mobile genetic elements (e.g., plasmids, integrons) ensure not only the co-transfer of these resistance traits, but also contributes to the persistence of resistance in the lack of antimicrobial pressure (Sandvang and Aarestrup, 2000).

In this genetic exchange both commensal and pathogenic bacteria are equally involved, moreover the bacterial host species barrier could also be crossed (Sørum and Sunde, 2001). Thus, the multifactorial nature of gene transfer creates particular problem and controversies, when the origin and the direction of transmission of specific resistance traits have to be established.

#### Integrons as important tools in the transfer of co-resistance

Integrons characteristically constitute small genetic systems possessing the ability to capture and co-express a set of resistance determinants with different functions. Integrons are ranked in four classes based on the homology of the integrase protein, among them class 1 integrons being the most frequently identified ones among *Enterobacteriaceae* including both commensal and pathogenic populations of *E. coli*. In the classical structure of the class 1 integron, antibiotic resistance gene cassettes are integrated sequentially in the variable region (VR), flanked by two conserved segments, 5′CS and 3′CS. The 5′CS contains the integrase gene (*intI1*), the promoter sequence, and an insertion site (*attI*) for various resistance gene cassettes, while the characteristic *qacEΔ1* and *sul1* genes are consequent in the 3′CS (Carattoli, 2001). Embedded gene cassettes lack their own promoter, therefore a common promoter is needed for their expression, which is located downstream of the integrase gene. In this manner a number of resistance genes coding diverse mechanisms can be clustered into the same expression unit, which provides a double benefit to the bacterial host: achieving MDR and reducing its biological cost at the same time (Guardabassi and Courvalin, 2006).

The mobilization of class 1 integrons is related to mobile genetic elements. In this respect transposon *Tn*21 is known to contribute to their mobility, moreover the physical association of integrons with IncF1 plasmid was also demonstrated (Carattoli et al., 2001). Associations of genes conferring resistance against several important antimicrobial classes, such as aminoglycosides, chloramphenicol, penicillins, trimethoprim, and sulfonamides are frequently carried by integrons. Interestingly, different antimicrobial classes are only moderately represented by resistance genes in different integron structures, suggesting that a restricted number and type of gene cassettes can be integrated. According to this, molecular characterization of *E. coli* strains reveal that class 1 integrons are working mostly with the following sets of genes: *aadA*-like, *aacA*-like and/or *aadB* (aminoglycoside), *catB*-like (chloramphenicol), *dfrA*-like (trimethoprim), whereas *sul1* (sulfamethoxazole) is conserved in the structure of class 1 integron.

Although the number and the subtype of the integrated gene cassettes may vary between animal species and sample sources (Nógrády et al., 2006; Karczmarczyk et al., 2011), no remarkable differences were detected, when the genetic diversity of integrons derived from healthy and sick animals were compared with those from humans (Cocchi et al., 2007). However, it was established, that the prevalence of integrons in MDR *E. coli* strains derived from animal fecal samples was higher than in human strains (Ho et al., 2009). This was in harmony with the results of one of the above studies from chicks (Nógrády et al., 2006), where the variability of class 1 integrons was higher among commensal strains as compared to extraintestinal pathogens. Some of these integrons were carried by conjugative plasmids, indicating the role of commensal *E. coli* as a reservoir of multiple resistance determinants. It is interesting to note, that class 1 integrons of 1.6 and 1.8 kb were detected in 22% of commensal *E. coli* isolates from farmed catfish, containing resistance gene cassettes *dfrA12-aadA2* and *dfrA17-aadA5*, respectively, beside the predominant tetracycline resistance genes *tet*(B) (77%), and *tet*(A) (25%), respectively (Nawaz et al., 2009).

On the other hand, such class 1 integrons of commensal *E. coli* bacteria could also be derived from *Salmonella*, further supporting the concept about *E. coli* bacteria as reservoirs of multiple resistance determinants (van Essen-Zandbergen et al., 2009). Time-related analysis of the integron carriage showed no variations when considering the overall relative frequency of integron prevalence in pig and in chicken strains from 1998/1999 and 2006, respectively (Marchant et al., 2013), although the molecular screening of the reference ECOR collection (1973–1980) revealed the presence of class 1 integrons in only four strains from the total of 72 reference *E. coli* strains tested (Mazel et al., 2000).

#### Plasmids as the most effective vectors in transferring multiresistance

Plasmids are self-replicating extra-chromosomal elements promoting the simultaneous inter- and intra-specific mobilization of genetic determinants, thereby being the most efficient tools in the acquisition and dissemination of antimicrobial resistance between bacteria of *Enterobacteriaceae*. Plasmids are able to accumulate a great variety of transposable elements, including transposons and insertion sequences that mobilize the antimicrobial resistance genes for transfer to the new host bacteria.

There are two ways in which bacteria can operate with their plasmid-borne array of resistance genes: (i) by distributing individual genes to several different plasmids and/or (ii) by clustering multiple genes into the same transfer unit, called MDR plasmid. Such MDR plasmids are often a result of interplasmidic recombination, integration of transposons, and/or insertion of resistance gene cassettes (Schwarz et al., 2006). The selective advantage provided by the physical association of multiple resistance genes when antimicrobials are administered in combinations, may explain the worldwide increasing trend of MDR in *E. coli* strains from animals and humans.

Plasmids mediating MDR are characterized as usually large (>50 kb), self-conjugative vehicles, with low copy number, and encode resistance to all key antimicrobial classes, including aminoglycosides, β-lactams, phenicols, quinolones, tetracyclines, and sulfonamides (Carattoli, 2013). Due to their extreme flexibility in the acquisition and transmission of the vast majority of resistance genes, plasmids may function as one kind of warning systems in the estimation of the current status of resistance determinants. The introduction and extended use of “new generation” antimicrobials in human and veterinary medicine to treat serious infections, resulted in the development and successful spread of “new generation plasmids,” serving as reservoirs among others of resistance genes such as CTX-M type ESBL, PMQR and carbapenemases. As an example, currently (date: 04/10/2013) some carbapenemase resistance plasmids of enterobacteria seem to generate serious epidemic concerns, especially for nursing homes and hospitals in the USA (). To our knowledge, to date there are no reports on the presence of carbapenemase producing commensal *E. coli* in food animals.

The widespread use of critically important antimicrobial agents including cephalosporins (third and fourth generation) and (fluoro)quinolones has triggered a rising incidence of these novel plasmid-mediated mechanisms in clinical isolates of enterobacterial species (Livermore, 2012). Surveillance reports on monitoring resistance phenotype in commensal *E. coli* from food animals indicated that resistance to newer compounds (cefotaxime and cefepime) was rarely or not detected in the European Union (European Food Safety Authority and European Centre for Disease Prevention and Control [EFSA and ECDC], 2010).

The extensive molecular characterization of resistance plasmids in *E. coli* strains of clinical interest allowed an insight to the underlying plasmid-mediated, usually MDR mechanisms, and the question can be raised, to what extent commensal strains of *E. coli* could be involved in the spread of such plasmids? Unfortunately, in case of commensal *E. coli* the significance of such MDR plasmids is merely estimated so far based on the identified resistance associations which are known to be carried by plasmids, and there is a need for more in depth molecular analysis.

The following sub-chapters intend to provide a brief summary of the current knowledge on the incidence of these critically important and novel resistance mechanisms and their plasmids in *E. coli* from healthy food animals, with special regard to those carrying specific gene associations.

***Extended-spectrum β-lactamase plasmids in commensal *E. coli* from food animals.*** The veterinary use of extended-spectrum β-lactams (third and fourth generation cephalosporins) over the last two decades, resulted in the emergence of plasmids carrying ESBLs (inactivators of the drug by hydrolysis) in *E. coli* strains of animal origin (Aarestrup, 2006). However, ESBL producing bacteria are reported overwhelmingly from human clinical cases (Jacoby and Munoz-Price, 2005). Li et al. (2007) provided a chronological report on ESBL-producing commensal *E. coli* of food animals between 1992 and 2005. Data suggested a worldwide distribution of several CTX-M type ESBL (*bla*_CTX-M_) variants (CTX-M-1, CTX-M-2, CTX-M-3, CTX-M-9, CTX-M-13, CTX-M-14, CTX-M-18, and CTX-M-24) in commensal *E. coli* strains among others from poultry, swine, and cattle.

Recently, several new CTX-M variants have been detected on conjugative plasmids, with *bla*_CTX-M-14_ and *bla*_CTX-M-15_ most frequently represented among *E. coli* strains from healthy pigs and poultry. Transfer experiments and plasmid replicon typing revealed significant diversity among CTX-M conjugative plasmids, with IncFII and IncI1 replicons predominantly detected. Among these, plasmids carrying *bla*_CTX-M-14_ seemed to be epidemic among healthy farm animals, contributing essentially to the dissemination and transfer of β-lactam resistance in China (Zheng et al., 2012).

Some of the CTX-M plasmids are often regarded as large MDR plasmids, encoding co-resistance and may provide broad conjugational host range. As an example, the fully sequenced plasmid pC15-1a of *E. coli* associated with the internationally acknowledged outbreak lineage ST131, encoding additional resistance to non-β-lactam antibiotics, including aminoglycosides, tetracycline, chloramphenicol, nalidixic acid, and sulfamethoxazole. The MDR region of this plasmid shows high similarity to the resistance region of the *bla*_CTX-M-15_ plasmid (pUUH239.2) of a recently described hospital outbreak strain of *Klebsiella pneumoniae* in Sweden (Sandegren et al., 2012). Three CTX-M-1 type IncN plasmids with co-resistance phenotypes to phenicols, spectinomycin, sulfamethoxazole, and tetracycline have been isolated from a pig farm in Denmark, which seemed to be capable to circulate between animals and humans (Moodley and Guardabassi, 2009). On the other hand, the comparative subtyping of CTX-M-1 plasmids with IncI1 replicons from avian and human *E. coli*, revealed no plasmids shared between *E. coli* of animal and human origin (Accogli et al., 2013), suggesting that elucidation of plasmid exchange and host specificity may need further investigations.

Moreover, animal derived CTX-M genes are also found to co-exist with other β-lactamases, including CMY-type AmpC β-lactamases (cephamycinases), which associations are particularly concerned, having even more serious animal and human health implications. The co-existence of *bla*_CMY-2_ with *bla*_CTX-M-1_, or *bla*_CTX-M-2_ and *bla*_CTX-M-55_ was reported in healthy chickens of the same farms, and occasionally in the same *E. coli* strains in Japan, although their presence on the same plasmids was not elucidated (Kameyama et al., 2013).

However, the CMY plasmids appear to always mediate multiple resistance associated with transposons and/or integrons (Li et al., 2007). Hiki et al. (2013) found that the conjugative transfer of CMY-2 MDR plasmids is associated to the IncA/C replicon type. Sequence analysis of multiresistant IncA/C plasmids from commensal and pathogenic *E. coli* derived from different animal sources reveal a remarkably stable and conserved plasmid backbone, which allow the acquisition of multiple resistance genes (Fernández-Alarcón et al., 2011). This broad host range nature of IncA/C plasmids may be an explanation to the worldwide distribution of *bla*_CMY-2_ genes as well.

In addition to above discussed associations, the co-carriage of *bla*_CTX-M-14_ with *bla*_CTX-M-15_, *bla*_OXA-1_, and *bla*_TEM-1_ (ampicillin) *floR* (phenicol), *tet*(A) (tetracycline), and *qnrS1* (fluoroquinolone) was found in *E. coli* isolates from pig (Huang et al., 2012). Although it was isolated from sick animals, the contamination of the healthy population of animals with this highly MDR plasmid may be expected.

***Plasmid-mediated quinolone resistance crossing animal/human barriers.*** It has been established that the introduction and worldwide use of fluoroquinolones in the treatment of enterobacterial infections has lead to the occurrence of (fluoro)quinolone resistance, both in the human- and veterinary medicine, showing increasing level and frequency also in *E. coli* strains from food animals (Webber and Piddock, 2001). The prime mechanisms of resistance to quinolones (mutational modification of DNA gyrase and topoisomerase IV, decreased outer membrane permeability and overexpression of innate efflux pumps) are chromosomally encoded, and thus are not regarded as being transferable.

In the last two decades, however, the plasmid-mediated resistances to (fluoro)quinolones (PMQR) are raising concerns as transferable mechanisms in human and in veterinary enterobacterial isolates (Poirel et al., 2012). Plasmid-mediated resistance to quinolones is partially due to the presence of the transferable Qnr proteins encoded by several variants of corresponding genes *qnrA*, *qnrB*, *qnrS*, *qnrC*, and/or *qnrD*. Above proteins protect the DNA gyrase and topoisomerase IV enzymes from the inhibitory activity of quinolones, conferring low-level resistance per se but facilitate the selection of highly resistant bacterial strains (Martínez-Martínez et al., 2008).

Among them the *qnrS1* gene variant seems to be the most frequently reported and worldwide diffused in both animal and human sources (Strahilevitz et al., 2009), and an increasing number of information has been recently accumulated on plasmids of *E. coli* strains carrying the *qnrS1* gene (Yue et al., 2008; Cerquetti et al., 2009; Huang et al., 2009; Ma et al., 2009; Xia et al., 2010; Szmolka et al., 2011), especially in poultry and pigs in Europe and in China. Besides, *qnrB* positive isolates of commensal *E. coli* have been reported from turkey samples in Europe (Veldman et al., 2011). Plasmids mediating *qnr*-type resistance often harbor other resistance genes conferring resistance to β-lactams, aminoglycosides, chloramphenicol, and tetracycline (Poirel et al., 2012).

In the transmission of the *qnrS1* determinant the IncN plasmids are frequently involved as described for human clinical isolates of *Salmonella enterica* (Hopkins et al., 2007; García-Fernández et al., 2009) and more recently for *E. coli* (Karah et al., 2010). The first evidence for the detection of *qnrS1* on IncN plasmids in *E. coli* strains of animal origin has been described for a MDR commensal porcine *E. coli* by Szmolka et al. (2011). Interestingly, *qnrS1* gene was identified in association with a *Tn3*-like transposon, similarly to that of *Salmonella* Infantis isolated from chicken (Kehrenberg et al., 2006). These observations, together with those of Guerra et al. (2003b) are further indicating that the transferability of resistance determinants from commensal *E. coli* of animals to humans could happen in different ways and none of these should be ignored.

As a further novel example of PMQR, the modified aminoglycoside acetyltransferase AAC(6′)-Ib-cr enzyme, capable for the enzymatic inactivation of ciprofloxacin, should be mentioned. This type of PMQR has been reported for commensal *E. coli* of poultry and swine origin in Tunisia and in China, respectively (Liu et al., 2011; Soufi et al., 2011), but also for *Salmonella* paratyphi B of chicken origin in China (Du et al., 2012), further supporting the evidences for inter-specific exchange of resistance determinants between commensal *E. coli* and zoonotic bacteria known to colonize humans.

Further interesting examples of PMQR are the plasmid-mediated quinolone efflux pump QepA and the MDR efflux pump OqxAB. Both have been detected in commensal *E. coli*. The *qepA* gene seem to be quite prevalent in swine in China, and shows a strong linkage with the high level aminoglycoside resistance determinant *rmtB *(Liu et al., 2008). Furthermore in Nigeria a commensal *E. coli* strain of chicken origin, simultaneously possessing *qepA *and* qnrB* genes have been detected (Fortini et al., 2011). The MDR efflux pump OqxAB has been detected in *E. coli* from swine manure in Denmark (Hansen et al., 2005), encoded on a conjugative plasmid pOLA52 (Norman et al., 2008) that conferred resistance to a veterinary growth promoter olaquindox, and later such OqxAB positive strains have been identified in commensal *E. coli* from chicken and swine from China (Liu et al., 2008; Zhao et al., 2010).

All these data are indicating that PMQR genes are globally prevalent in the commensal *E. coli* strains, especially of poultry and pigs and they seem to be trafficking between *Salmonella* and *E. coli* thereby having an access to the human intestinal flora and gaining a possible worldwide clinical significance in man.

## CONCLUDING REMARKS AND FUTURE PROSPECTS

### ADVANTAGES AND DISADVANTAGES OF GENETIC FLEXIBILITY OF COMMENSAL *E. coli*

The fact that *E. coli* is one of the genetically and metabolically most flexible organisms of the normal intestinal flora is also evidenced in its responses to antimicrobial therapy. This remarkable adaptability of commensal *E. coli* to toxic environments as resulted by antimicrobial interventions can primarily be attributed to its effective efflux systems. These systems are the first defense tools of *E. coli* and thus they can be regarded as part of their innate resistance mechanisms to antibiotics. As a result, these bacteria adapt to the newly introduced antimicrobials (e.g., streptomycin or tetracycline) within hours after treatment, and respond by an increased population that may become dominant in the intestinal flora for a few days and – depending of the antimicrobials used – may remain in minority of the normal *E. coli* flora later on (Videnska et al., 2013). In case of fluoroquinolone treatment, however, the chromosomally encoded resistance appeared and remained characteristic to the normal *E. coli* flora of chicks (Barrow et al., 1998). Similar persistence of CTX-M producing *E. coli* was observed in case of application of β-lactam antibiotics in pigs due to acquired CTX-M genes (Cavaco et al., 2008). Thus, such commensal *E. coli* becoming resistant, seem to help maintaining the physiological balance of the normal intestinal flora in spite of application of antimicrobials.

The genetic flexibility of *E. coli* not only ensures successful survival and growth under adverse environmental conditions, but also makes these bacteria to be very effective recipients and even distributors of newly introduced foreign genes, such as antimicrobial resistance determinants, and associated mobile genetic elements (plasmids, transposons, and integrons) through horizontal gene transfer. Thereby commensal *E. coli* also have a great potential as a reservoirs for antimicrobial resistance. The above versatility and genetic flexibility are enabling of *E. coli* to develop resistances against multiple antimicrobial classes.

In short, commensal *E. coli* flora can be regarded as a rich source of emerging and spreading antimicrobial resistant strains or resistance determinants (da Costa et al., 2013). On the other hand the antimicrobial resistance of commensal *E. coli* flora to antimicrobials can be an advantage by helping to keep the microbiological–physiological balance of the large intestine during and after antimicrobial treatments.

### ORIGIN AND EMERGENCE OF ANTIMICROBIAL RESISTANCE DETERMINANTS IN *E. coli* FROM ANIMALS

Regarding chromosomal- and mobile determinants of antimicrobial resistance, commensal *E. coli* strains do not seem to be essentially different from pathogenic counterparts (Nógrády et al., 2006; Szmolka et al., 2012). In fact the huge genetic versatility of commensal *E. coli* populations ensures an elevated potential as a reservoir for several classical and new or even unknown and/or undetected resistance determinants (plasmids, transposons, integrons) and other transposable genetic elements, such as the recently discovered insertion sequence common region (ISCR) elements, probably emerging as a response to new antimicrobials (Call et al., 2010; Iyer et al., 2013). Such emergence may even be more frequently observed in geographical areas and clinical practices where the use of antimicrobials is more intense and/or less prudent. There are observations indicating that the presence of some widely used antibiotics may drive the evolutionary mechanisms of *E. coli* on a higher speed through SOS response, inducing integrase transcription and increased recombination of resistance gene cassettes (Baharoglu et al., 2010; Cambray et al., 2011).

In some cases, the origin of several resistance determinants may be found in the aquatic environments as indicated by the example of the *qnr* type PMQR genes (Poirel et al., 2012) and in the soil, as exemplified for fluoroquinolone and several other resistances in soil-dwelling bacteria (Dantas et al., 2008). In fact, the environment in general could be regarded as a melting pot of antimicrobial resistance (da Costa et al., 2013).

The extensive molecular characterization of resistance in *E. coli* strains of clinical interest allowed better insight to some underlying plasmid-mediated mechanisms as well. In contrast to clinical strains, plasmids from commensal strains of *E. coli* from various sources are much less investigated. Their presence is only hypothetical in most of the studies, and is based on the identified co-resident pheno- and/or genotypes known to be associated with plasmids and plasmid-associated mobile genetic elements (Johnson and Nolan, 2009).

### PUBLIC HEALTH CONSIDERATIONS

As indicated in Section “Multidrug Resistance in Commensal *E. coli*”, the public health significance of antimicrobial resistances of commensal *E. coli* can be much greater then it is generally assumed today. Here we should take into account that a regular antimicrobial therapy of animals or man could quickly select MDR populations of* E. coli* in different group of animals. These nascent MDR strains will have a great chance to propagate in the newly opened biological niche, resulted by the elimination of other competing members of the intestinal flora.

The other important factor in predicting the public health significance of antimicrobial resistance of commensal *E. coli* is, that their resistance genes could be efficiently transferred *in vivo* to pathogenic strains of *E. coli* or to *Salmonella* and vice versa as indicated by molecular epidemiological data (Nógrády et al., 2006; Szmolka et al., 2011). This can be explained by assuming that the MDR population of *E. coli* is not only becoming dominant in the intestine but – as a result of possible antimicrobial interference and host response – will start disseminating its versatile mobile genetic vectors, most often conjugative plasmids for antimicrobial resistance, or for increase fitness or virulence (Bonnet et al., 2009). On the other hand, this dominant commensal *E. coli* flora also offers an exponentially increasing pool of diverse, and potentially recipient bacteria for the horizontal transfer of mobile genetic elements, carrying antimicrobial resistance and/or virulence genes (O’Brien, 2002; Summers, 2002). As exemplified by the *Salmonella* Typhimurium model of Stecher et al. (2012), this horizontal gene transfer to commensal *E. coli* can be enhanced by the pathogen driven intestinal inflammatory response of the host organism, greatly facilitating the conjugative transfer and reassortment of plasmid encoded genes.

With the above options in mind, the question about human colonization potential of animal commensal *E. coli* cannot be avoided. According to the review of Hammerum and Heuer (2009) there are several examples of humans colonized by antimicrobial resistant commensal *E. coli* from food animals, thereby presenting antimicrobial resistance burdens, possibly limiting therapeutic options.

There are, however, examples of genes confirming resistance to aminoglycosides (*aac(3)-I*, *ant(2″)-Ia* and *aac(6′)-Ib*), or to chloramphenicol (*catB*) occurring almost exclusively in human *E. coli* in contrast to animal isolates (Szmolka et al., 2012). Similarly, Ewers et al. (2012) suggested that the animal ESBL producing *E. coli* did not seem to be a major source for human ESBL strains. Both examples indicate certain differences between MDR *E. coli* of animal and human origin.

Farm workers in animal production areas represent a special group in this respect. They are more prone to contamination by MDR *E. coli* of animal origin and they become more frequently carriers of MDR *E. coli* from animals (van den Bogaard et al., 2001), while raw foods can also be a frequent source of human contamination (Musgrove et al., 2006; Zhao et al., 2012; da Costa et al., 2013). As a further example for public health significance of *E. coli* resistance determinants of animal origin was provided by Moodley and Guardabassi (2009) by detecting the same or very similar CTX-M plasmids of IncN type across multiple *E. coli* lineages between farm workers and pigs. It seems, that future studies should be directed to quantify and characterize microbial risks derived from commensal *E. coli* strains from food animals as potential contaminants to man and/or “active reservoirs” of specific resistance genes or of MDR determinants transferable to human.

Consequently, due to the constantly changing nature of resistance, monitoring of antimicrobial attributes (pheno- and genotypes) of normal intestinal *E. coli* (and *Enterococcus*) from food animals is a necessary and important measure to assess ongoing trends, and thereby keeping the national and community services informed on actual developments on the area of antimicrobial resistances and their determinants. Such combined pheno- and genotypic characterizations, together with appropriate gene expression-, and metagenomic studies would better highlight the importance of commensal *E. coli* of food animals, as a so far less recognized and much less appreciated reservoir of multiple antimicrobial resistance mechanisms.

## Conflict of Interest Statement

The authors declare that the research was conducted in the absence of any commercial or financial relationships that could be construed as a potential conflict of interest.
